# Body Dissatisfaction Revisited: On the Importance of Implicit Beliefs about Actual and Ideal Body Image

**DOI:** 10.5334/pb.362

**Published:** 2018-01-04

**Authors:** Niclas Heider, Adriaan Spruyt, Jan De Houwer

**Affiliations:** 1 Department of Experimental Clinical and Health Psychology, Ghent University, BE

**Keywords:** Implicit measures, relational responding task, body dissatisfaction, eating disorder

## Abstract

Body dissatisfaction (i.e., a negative attitude towards one’s own physical appearance) is assumed to originate from a perceived discrepancy between the actual physical appearance (i.e., actual body image) and the desired ideal state of the body (i.e., ideal body image). We assessed implicit beliefs about these two aspects of the body image independently using two Relational Responding Tasks (RRT) in a sample of participants who were either low or high in explicitly reported body dissatisfaction. As hypothesized, differences in body dissatisfaction exerted a differential influence on the two RRT scores. The implicit belief that one is thin was less pronounced in participants who were strongly dissatisfied with their body relative to participants who were more satisfied with their body. The implicit desire to be thin (i.e., thin ideal body image), in contrast, tended to be more pronounced in participants who exhibited a high degree of body dissatisfaction as compared to participants who exhibited a low degree of body dissatisfaction. Hierarchical regression analyses also revealed that the RRT scores were predictive of self-reported body dissatisfaction, even over and above the predictive validity of some (but not all) explicit predictors of body dissatisfaction that were included in the present study. More generally, these findings contribute to the empirical validation of the RRT as a measure of implicit beliefs in the context of body dissatisfaction.

Body (image) dissatisfaction can be defined as the negative attitude towards one’s own body resulting from a perceived discrepancy between the actual body image (i.e., perceptions, thoughts, and feelings concerning one’s actual physical appearance; e.g., Cash, 1990) and the ideal body image (i.e., internalized ideals about one’s physical appearance; e.g., Cooper & Taylor, 1988; Strauman, Vookles, Berenstein, Chaiken, & Higgins, 1991; Williamson, Gleaves, Watkins, & Schlundt, 1993). Body dissatisfaction has been identified as one of the key factors for dieting behavior, negative affect, and the causation and maintenance of eating disorders (American Psychiatric Association, 2013; Fairburn & Harrison, 2003; Stice, 2001, 2002). Accordingly, over last few decades, behavioral scientists have invested a great deal of effort in the development of measures of body dissatisfaction (Allebeck, Hallberg, & Espmark, 1976; Bessenoff & Sherman, 2000; Bluemke & Friese, 2012; Degner & Wentura, 2009; Freeman, Thomas, Solyom, & Hunter, 1984; Heider, Spruyt, & De Houwer, 2015; Juarascio et al., 2011; Parling, Cernvall, Stewart, Barnes-Holmes, & Ghaderi, 2012; Roddy, Stewart, & Barnes-Holmes, 2010, 2011; Slade & Russell, 1973). Most of these instruments, however, are self-report measures (i.e., questionnaires) and are thus limited in two ways. First, it is well-known that self-report measures are susceptible to effects of social desirability and impression management (Cronbach, 1990; Holtgraves, 2004). For example, when completing a body-dissatisfaction questionnaire, anorectic patients may be motivated to respond untruthfully because of far-reaching therapeutic consequences (e.g., compulsory admission). Second, as discussed by Greenwald and Banaji (1995), self-report measures are, by definition, unsuited to capture traces of past experiences that are introspectively unidentified (e.g., the accidental but repeated exposure to the thin ideal on a social media).

To resolve both problems, behavioral scientists have started to develop diagnostic instruments that allow for an assessment of psychological constructs (e.g., attitudes, biases, beliefs) without relying on self-reports. More specifically, these ‘implicit measures’ aim at capturing inter-individual differences under automaticity conditions, for example by capitalizing on the well-known principle of automatic response facilitation/interference, the presentation of construct-relevant stimuli under subjective recognition thresholds, and/or the implementation of strict response deadlines (for an extensive discussion of the meaning of the concept ‘implicit measure’, see De Houwer, Teige-Mocigemba, Spruyt, & Moors, 2009).

Roughly, the class of implicit measures can be divided into two broad subclasses. Associative implicit measures, like the (standard) evaluative priming task (EPT; Fazio, Jackson, Dunton, & Williams, 1995), the Implicit Association Test (IAT; Greenwald, McGhee, & Schwartz, 1998), or the Affect Misattribution Procedure (AMP; Payne, Cheng, Govorun, & Stewart, 2005), are designed to assess the associative strength between concepts in memory (Hughes, Barnes-Holmes, & Vahey, 2012). Associative implicit measures can, for instance, be used to capture the extent to which a certain class of stimuli (e.g., spiders) is associated with a positive or negative valence.

In many cases, however, it is not only important to examine to what extent two concepts are related in memory but also the precise way in which they are related (e.g., Heider et al., 2015; Remue, De Houwer, Barnes-Holmes, Vanderhasselt, & De Raedt, 2013; Remue, Hughes, De Houwer, & De Raedt, 2014). In these cases, the use of relational implicit measures, like the Implicit Relational Assessment Procedure (IRAP; Barnes-Holmes et al., 2006) and the Relational Responding Task (RRT; De Houwer, Heider, Spruyt, Roets, & Hughes, 2015) is more appropriate. These measures were designed specifically to allow for an assessment of the way in which individuals tend to relate two stimuli to one another automatically. In the context of spider fear, for example, respondents who complete an RRT may be presented with relational information concerning spiders (e.g., the statements ‘I like spiders’ and ‘I hate spiders’). Their task is to respond ‘true’ or ‘false’ on the basis of an instructed response rule. For example, participants may be asked to respond as if they despise spiders in one block of trials (e.g., respond ‘true’ to the statement ‘I hate spiders’) and to respond as if they like spiders in a second block of trials (e.g., respond ‘false’ to the statement ‘I hate spiders’). By examining which relational information (e.g., ‘like’, ‘hate’) results in optimal task performance given a specific response rule, one can learn about the precise way in which respondents tend to relate specific stimuli (e.g., ‘I’, ‘spiders’). Moreover, given that participants are required to respond as fast as possible while acting *as if* they endorse a certain belief, it can be argued that RRT scores are more implicit in terms of speed and unintentionality than traditional explicit measures such as questionnaires (for an extensive discussion of the ‘implicit’ nature of the RRT, see Heider et al., 2015).

As argued above, body dissatisfaction depends on beliefs about the actual and the ideal body image. Crucially, these two types of body image differ only in how the concepts ‘self’ and ‘body-size’ are related to another. Specifically, beliefs about the actual state of one’s body involve a descriptive relation (e.g., ‘I am thin’) whereas beliefs about the ideal state of one’s body involve a relation of desirability (e.g., ‘I want to be thin’). Therefore, relational implicit measures should be best suited to assess these different beliefs at the implicit level.

Preliminary evidence for this assertion was recently published by Heider et al. (2015). In their study, participants completed two IRAPs, one to capture actual body image (hereafter referred to as actual-IRAP) and one to capture ideal body image (hereafter referred to as ideal-IRAP). In both tasks, two stimuli were presented on each trial. The so-called sample stimuli (i.e., ‘I am’ vs. ‘I am not’) were presented at the top of the computer screen. The so-called target stimuli (i.e., words referring to thinness and overweight) were presented in the center of the computer screen. Crucially, the combinations of the two stimuli were either congruent or incongruent with being thin (e.g., ‘I am’ + ‘skinny’ and ‘I am’ + ‘chubby’, respectively). In one set of trials, participants were asked to respond as if they believed themselves to be thin by selecting the appropriate response option (i.e., ‘true’ or ‘false). In as second set of trials, they were asked to respond as if they believed themselves to be overweight. The ideal-IRAP was identical to the actual-IRAP except for the fact that participants were (a) presented with stimulus combinations that reflected the desire to be thin or overweight (e.g., ‘I want to be + skinny’ and ‘I want to be + chubby’, respectively) and (b) required to respond as if they desired to be thin or overweight in different sets of trials. Based on the definition of body dissatisfaction as the negative attitude towards one’s own body resulting from the perceived discrepancy between actual and ideal body image, Heider et al. (2015) expected the scores of actual-IRAP and ideal-IRAP to vary as a function of self-reported body dissatisfaction. As anticipated, results indicated that the belief to be thin (as measured by the actual-IRAP) was more pronounced in participants who were low in body dissatisfaction as compared to participants who were high in body dissatisfaction. Scores of the ideal-IRAP, in contrast, revealed that the desire to be thin was less pronounced in participants low in body dissatisfaction as compared to participants high in body dissatisfaction. These observations support the validity of the IRAP as a measure of specific body-related beliefs.

In the present report, we revisit the implicit measurement of ideal and actual body image, for two reasons. First, the IRAP is a challenging task to complete, even in respondents who are highly familiar with computerized (reaction-time) tasks. The first phase of an IRAP typically consists of a number of practice blocks in which participants are required to reach a certain threshold in terms of average speed (typically between 2000 ms and 5000 ms) and accuracy (typically between 65 % and 80 %, for a review, see Hughes & Barnes-Holmes, 2013). After the practice phase, participants can continue with the actual test phase of the IRAP only if both criteria are met. Compared to the normal error rates and average response speed in the associative implicit measures, these criteria are rather liberal. Still, attrition rates of 20% or more are common in IRAP research (e.g., Remue et al., 2013; see Hughes & Barnes-Holmes, 2013, Table 1, for an overview). Clearly, if it is to be of value outside of the laboratory, implicit measures of ideal and actual body should preferably be less demanding to complete than the standard IRAP.

**Table 1:** Correlations between measures.

		*M*	*SD*	*min*	*max*	1	2	3	4	5	6	7	8

1	Body Dissatisfaction (EDI)	33.17	14.58	12	54	–	0.54***	–0.36**	0.28*	0.67***	–0.06	–0.85***	0.80***
2	BMI	21.71	3.17	17.26	31.02		–	–0.34**	0.00	0.82***	0.48***	–0.76***	0.62***
3	actual-RRT	0.08	0.30	–0.46	0.71			–	0.01	–0.28*	–0.10	0.30**	–0.15
4	ideal-RRT	–0.05	0.33	–0.63	0.63				–	0.03	–0.36**	–0.22^+^	0.13
5	actual-CDRS	4.98	1.69	2	9					–	0.45***	–0.80***	0.70***
6	ideal-CDRS	3.61	0.97	2	6						–	–0.07	–0.04
7	Statements (Actual-RRT)	2.98	1.27	1	5							–	–0.85***
8	Statements (Ideal-RRT)	4	0.83	1.2	5								–

*Note*. actual = actual body image; ideal = ideal body image; RRT = Relational Responding Task; CDRS = Contour Drawing Rating Scale; EDI = Body dissatisfaction subscale of the Eating Disorders Inventory; BMI = Body Mass Index. ^+^*p* < .10; *p < .05; **p < .01; ***p < .001.

As second point of concern, one may note that the reliability of the IRAP is limited. In a review of 27 IRAP studies (Hughes & Barnes-Holmes, 2013), good internal consistency estimates were observed only if the training criteria for the practice blocks were quite liberal (i.e., mean response latencies between 3000 ms and 5000 ms, error rates between 65 % and 70 %; e.g., Barnes-Holmes, Murtagh, Barnes-Holmes, & Stewart, 2010; Campbell, Barnes-Holmes, Barnes-Holmes, & Stewart, 2011; Drake et al., 2010). This observation may be problematic because social desirability and impression management concerns are more likely to affect task performance as participants are given more time to respond on each individual trial.

Studies using the RRT (e.g., De Houwer et al., 2015), on the other hand, suggest that the RRT is user-friendly (i.e., attrition rates as low as 5%, mean response times well below 2000 ms, and error rates well below 20 %) whilst its reliability is still acceptable (e.g., *Rsb* = 0.64 in De Houwer et al., 2015). Accordingly, in the present study we re-addressed the implicit assessment of body dissatisfaction by using the RRT. To capture beliefs about actual body image, participants were presented with statements like ‘I possess a slim body’ and ‘I see myself as a fat person’ (hereafter referred to as the actual-RRT). To capture beliefs about ideal body image, participants were presented with statements like ‘I wish I was thinner’ and ‘I strive to weigh more’ (hereafter referred to as the actual-RRT). In line with Heider et al. (2015), we hypothesized that participants would differ in their RRT scores as a function of their level of explicit body dissatisfaction. More specifically, given that our sample consisted of young female adults who typically desire to be thin rather than overweight (i.e., thin-ideal internalization; e.g., J. K. Thompson & Stice, 2001), we expected the belief to be thin to be more pronounced in participants low in body dissatisfaction as compared to participants high in body dissatisfaction. In contrast, we expected the desire to be thin to be less pronounced in participants low in body dissatisfaction as compared to participants high in body dissatisfaction. Participants also completed a number of explicit measures of actual and ideal body image. First, they were asked to rate their endorsement of the statements used in the two RRTs. Second, they were asked to choose from a range of schematic body images the images that corresponded with their actual and ideal body (i.e., the Contour Drawing Rating Scale, CDRS, M. A. Thompson & Gray, 1995). As participants were healthy university students who were tested anonymously, we expected them to respond truthfully on the explicit measures. We thus expected implicit and explicit measures of actual and ideal body image to correlate.

## Method

### Ethics Statement

Participants gave written informed consent prior to their participation and received course credit (*n* = 62) or payment of €7 (*n* = 6) in exchange for their participation. The experiment was approved by the ethics committee of the Faculty of Psychology and Educational Sciences of Ghent University.

### Participants

At the beginning of the academic year, 468 students at Ghent University completed the body dissatisfaction subscale of the Eating Disorders Inventory (EDI; Garner, Olmstead, & Polivy, 1983) during an online screening study that involved multiple questionnaires. To ensure that our sample included participants who were either low or high in body dissatisfaction, we invited all female students who had scored within the first and fourth quartile of the total EDI distribution to participate in an individual lab session (N = 143). Note, however, that the invitation to participate in the experiment was sent anonymously via an online recrutement system. It was thus necessary to administer the EDI for a second time during the actual experimental session to identify group membership. In total, 68 female students (*M* = 18.72 years, *SD* = 2.12) responded to our invitation and participated. All participants were Dutch speakers and had normal or corrected-to-normal vision.

### Measures

#### RRTs

Participants completed two RRTs, one to capture implicit beliefs about actual body image (i.e., actual-RRT) and one to capture implicit beliefs about ideal body image (i.e., ideal-RRT). In line with De Houwer et al. (2015), two types of stimuli were used in both RRTs, inducer words and target statements. The same set of 10 inducer words was used in both RRTs, five of which were synonyms of ‘true’ and five of which were synonyms of ‘not true’ (see Table 2 in the Appendix). Two sets of 20 sentences were used as target statements, one set for each RRT. Target statements for the actual-RRT related the concepts ‘thin’ and ‘overweight’ to the concept ‘self’ in a descriptive way. Five statements referred to the belief to be thin (e.g., ‘I possess a slim body’) and five statements referred to the belief to be overweight (e.g., ‘I weigh too much’). Negations of these ten statements led to the creation of 10 additional target statements that referred to the belief not to be overweight (e.g., ‘I do not weigh too much’) and the belief not to be thin (e.g., ‘I do not possess a slim body’), respectively. Negations were included to ensure that participants would be required to process the meaning of the entire statement (and not just a subset of words) in order to respond correctly. For the ideal-RRT, the same target concepts were used, but now the target statements specified a relation of desirability. Five statements referred to the desire to be thin (e.g., ‘I desire to weigh less’) and five target statements referred to the desire to be overweight (e.g., ‘I strive to weigh more’). Again, negations of these ten statements were used as 10 additional target statements that referred to the desire not to be overweight (e.g., ‘I don’t strive to weigh more’) and the desire not to be thin (e.g., ‘I don’t desire to weigh less’; for the complete list of the target statements and their English translations, see Tables 3 and 4 in the Appendix).

In both RRTs, on each trial either one of the inducer words or one of the target statements was presented on the computer screen. Trials can therefore be divided into inducer trials and target trials. On all trials, participants were instructed to categorize the presented item as ‘true’ or ‘not true’ by pressing the right or left ctrl-key of the keyboard, respectively. It may be noted that the inducer trials were included to ensure that the responses were endowed with the meaning ‘true’ and ‘false’, similar to other implicit measures such as the Extrinsic Affective Simon Task (De Houwer, 2003).

Each RRT comprised seven blocks. In line with the typical IAT approach (Greenwald et al., 1998), three blocks were required to familiarize participants with the different response tasks (i.e., Blocks 1, 2, and 5). The remaining blocks (i.e., Blocks 3, 4, 6, and 7, sometimes also referred to as ‘mixed blocks’) were used for the actual assessments. In Block 1 (40 trials), each of the ten inducer words was presented four times. Participants were asked to classify these words as ‘true’ of ‘false’ as fast as possible. In Block 2 (40 trials), the 20 target statements were presented twice. Participants were asked to respond as if they were thin (in the actual-RRT) or wanted to be thin (in the ideal-RRT). For example, in the actual-RRT, the response ‘false’ was required when the statement ‘I weigh too much’ was presented. In blocks 3 and 4 (40 trials each), the ten inducer words were presented twice and the 20 target statements were presented once. Participants were asked to respond in accordance with the response rules practiced during the preceding blocks. Block 5 (40 trials) was identical to Block 2, except for a reversal of the response rule. Participants were now asked to respond as if they were overweight (in the actual-RRT) or wanted to be overweight (in the ideal-RRT). For example, in the ideal-RRT, the response ‘true’ was required when the statement ‘I strive to weigh more’ was presented. Blocks 6 and 7 (40 trials each) were identical to Block 3 and 4, but participants were asked to respond in line with the response rule practiced in Block 5. The order of the different items was random within each block, with the restriction that the same item could not be repeated on consecutive trials.

Each trial started with the presentation of an item (i.e., inducer word or target statement) in the middle of the computer screen in Tahoma letter font, 28-point font size. Inducer words were presented in white in both RRTs, whereas the target statements were presented in orange (actual-RRT) or blue color (ideal-RRT). Different colors were used to increase awareness of the fact that different target statements were presented in the two RRTs. Items remained on screen until the correct response was registered. Incorrect responses were signaled by the presentation of a red X (Arial, 72-point font size) below the item until participants gave the correct response. The next trial started 750 ms after registration of the correct response. The RRTs were presented on a 17-inch LCD screen (60 Hz, 1440x × 900 pixels) and were written in Affect 4.0 (Spruyt, Clarysse, Vansteenwegen, Baeyens, & Hermans, 2010).

#### Self-report measures

Participants rated each of the 40 target statements used in the RRTs on a 5-point scale, ranging from 1 (disagree completely) to 5 (agree completely). For each individual participant and each class of target statements, these ratings were aggregated to obtain an explicit measure of actual body image and ideal body image. Actual and ideal body image were also measured using the female version of the Contour Drawing Rating Scale (CDRS; M. A. Thompson & Gray, 1995). The CDRS consists of nine schematic (female) figures of varying sizes ranging from underweight (1) to overweight (9). Participants completed the CDRS twice, once to indicate their actual body image and once to indicate their ideal body image. Explicit body dissatisfaction was assessed by means of the body dissatisfaction subscale of the Eating Disorder Inventory (EDI, 9 items; Garner et al., 1983), which has excellent psychometric qualities (e.g., Clausen, Rosenvinge, Friborg, & Rokkedal, 2011). Finally, we computed the Body Mass Index (BMI) for each participant using self-reported weight and height. We did not collect objective measures of weight and height as we observed an almost perfect correlation between factual and self-reported BMI measures in prior research (i.e., *r* = 96, Heider et al., 2015).

### Procedure and Group Assignment

All participants were tested individually during an experimental session that on average lasted 35 minutes. Because the focus of the present research was on the usefulness of the actual-RRT and the ideal-RRT, we wanted to ensure that performance in these tasks was not influenced by the prior completion of explicit measures. Accordingly, the experimental session always started with the completion of the two RRTs (in a counterbalanced order). Subsequently, participants were asked to rate the target statements used in the two RRTs and to complete the EDI and CDRSs for actual and ideal body image. Finally, participants were asked to report their weight and height for the calculation of the BMI.

As sampling was conducted anonymously via an online recruitment system, group assignment was based on the EDI ratings collected during the actual lab session. Participants were assigned to either the low or the high body dissatisfaction group by means of a cluster analysis. In the low body dissatisfaction group (*n* = 31), the mean EDI score was 19.1 (*SD* = 4.6, min = 12, max = 32). In the high body dissatisfaction group (*n* = 33), the mean EDI score was 46.4 (*SD* = 5.2, min = 36, max = 54). Both groups differed significantly in terms of their mean EDI score, *t*(62) = 22.35, *p* < .001. There was no overlap between groups in terms of the EDI score. In comparison to the EDI data collected by Clausen et al. (2011), the mean level of body dissatisfaction observed in the low body dissatisfaction group was already slightly elevated relative to normal controls (i.e., 19.1 vs. 15.3). In the low body dissatisfaction group, the mean level of body dissatisfaction was very high, even compared to a sample of eating disorder patients (i.e., 46.4 vs. 27.9).

## Results

### Data Preparation

The data of two participants were excluded from the analyses because their mean reaction times in both tasks (2428 ms and 2629 ms, for the actual-RRT; 2862 ms and 2838 ms, for the ideal-RRT) exceeded our cutoff criterion of 2.5 standard deviations above the grand mean of the respective tasks (actual-RRT: *M* = 1292 ms, *SD* = 369 ms; threshold = 2215 ms; ideal-RRT: *M* = 1521 ms, *SD* = 417 ms; threshold = 2563 ms; see Ratcliff, 1993). We also excluded the data of two other participants whose error rates in one of the two tasks (i.e., 27.5 % and 35.0 %) exceeded the cutoff criterion of 2.5 standard deviations above the grand mean of that task (actual-RRT: *M* = 11.2 %, *SD* = 5.3 %; threshold = 24.4 %; ideal-RRT: *M* = 11.3 %, *SD* = 6.7 %; threshold = 28 %; see Ratcliff, 1993). The mean reaction time on target trials in the actual-RRT was 1284 ms (*SD* = 341 ms), with participants responding incorrectly on 10.9 % (*SD* = 4.8 %) of the target trials. In the ideal-RRT, participants needed, on average, 1517 ms (*SD* = 394 ms) to respond on target trials and the error rate was 10.8 % (*SD* = 6 %).

For each participant and each version of the RRT, the raw response latencies obtained in the diagnostic blocks (i.e., Blocks 3, 4, 6, and 7) were transformed into D scores using the D1 algorithm described by Greenwald, Nosek, and Banaji (2003). The D1 algorithm was chosen because reaction times were recorded until a correct response was detected, thus removing the need for the use of a penalty for incorrect responses. Following the guidelines of Greenwald et al. (2003), the calculation of the D1 scores involved two steps. First, both for the actual-RRT and the ideal-RRT, separate D1 scores were calculated for the first and the second half of the diagnostic blocks (i.e., Blocks 3/6 vs. Blocks 4/7). The mean response latencies observed in Blocks 3 and 4 were thus subtracted from the mean response latencies observed in Blocks 6 and 7, respectively, and each difference score was divided by the standard deviation of the respective response latencies. In a second step, for each RRT, an overall D1 score was computed by averaging the two D1 scores obtained for each pair of blocks. In sum, for the actual-RRT, the D1 scores were computed such that higher scores were indicative of the (implicit) belief to be thin. Similarly, for the ideal-RRT, the D1 scores were computed such that higher scores were indicative of a more pronounced (implicit) desire to be thin.

For the actual-RRT, D1 scores ranged from –0.46 to 0.71, with a mean score of *M* = 0.08 (*SD* = 0.30), which differed from zero, *t*(63) = 2.07, *p* = .043, *d* = 0.26. For the ideal-RRT, D1 scores ranged from –0.63 to 0.63, with a mean score of *M* = –0.05 (*SD* = 0.33), which did not differ from zero, *t*(63) = 1.33, *p* = .189, *d* = 0.17.

### Effects at the Group Level

#### RRTs

To investigate whether the RRT scores were dependent upon the degree of self-reported body dissatisfaction, the overall RRT scores were submitted to a 2 (RRT: actual vs. ideal) × 2 (body dissatisfaction: high vs. low) ANOVA. As expected, we found a significant interaction of body dissatisfaction and RRT, *F*(1, 62) = 8.80, *p* = .004, \eta _p^2 = .12. Participants low in body dissatisfaction scored higher on the actual-RRT than participants high in body dissatisfaction, 0.18 vs. –0.02, *t*(62) = 2.72, *p* = .008, *d* = 0.68. Conversely, in absolute terms, participants high in body dissatisfaction scored higher on the ideal-RRT than participants low in body dissatisfaction, 0.00 vs. –0.11, *t*(62) = 1.40, *p* = .166, *d* = 0.35. In addition, we found a significant main effect of RRT, *F*(1, 62) = 6.90, *p* = .011, \eta _p^2 = .10, indicating higher scores on the actual-RRT as compared to the ideal-RRT, 0.08 vs. –.05. No other effects were significant, all *F*s < 1, all *p*s > .475.

To examine the reliability of the RRTs, reliability coefficients were estimated using a bootstrap procedure. For each RRT and each of 100 random-splits of the data, the correlation across participants between the two RRT scores was calculated. Correlations were then averaged. This procedure resulted in spearman-brown corrected mean split-half correlations of *Rsb* = 0.49 and *Rsb* = 0.57, for the actual-RRT and the ideal-RRT, respectively.

#### Explicit ratings of the target statements of the RRTs

To investigate whether the explicit ratings of the target statements used in the RRTs were also dependent upon the degree of self-reported body dissatisfaction, they were used as dependent variables in a 2 (type of target statement: actual body image vs. ideal body image) × 2 (body dissatisfaction: high vs. low) ANOVA. Results mirrored those of the RRT scores. We found a significant interaction of type of target statement and body dissatisfaction, *F*(1, 62) = 135.09, *p* < .001, \eta _p^2 = .69, indicating that participants high and low in body dissatisfaction differed in their ratings of the two types of target statements. Specifically, the explicit endorsement of the belief to be thin was more pronounced in participants who were low in body dissatisfaction as compared to participants who were high in body dissatisfaction, 4.09 vs. 1.93, respectively, *t*(62) = 12.97, *p* < .001, *d* = 3.24. In contrast, ratings revealed that the explicit desire to be thin was more pronounced in participants who were high in body dissatisfaction as compared to participants who were low in body dissatisfaction, 4.55 vs. 3.42, respectively, *t*(62) = 7.46, *p* < .001, *d* = 1.86. Mimicking the RRT data, we also found a significant main effect of the type of statement, *F*(1, 62) = 47.83, *p* < .001, \eta _p^2 = .44. Overall, the explicit belief to be thin was more pronounced than the explicit desire to be thin, 4.00 vs. 2.97. Finally, we found a significant main effect of group, *F*(1, 62) = 49.30, *p* < .001, \eta _p^2 = .44, indicating that participants who were high in body dissatisfaction were on average more extreme in their rating of the target statements than participants who were low body in body dissatisfaction, 3.24 vs. 3.75.

#### CDRS scores

A 2 (type of CDRS: actual body image vs. ideal body image) x 2 (body dissatisfaction: high vs. low) ANOVA revealed a significant interaction between the type of CDRS and body dissatisfaction, *F*(1, 62) = 66.45, *p* < .001, \eta _p^2 = .52. In line with the results reported above, the mean score on the actual-CDRS was higher in participants who reported a high degree of body dissatisfaction as compared to participants who reported a low degree of body dissatisfaction, 6.03 vs. 3.87, respectively, *t*(62) = 6.59, *p* < .001, *d* = 1.65. In contrast, there was no evidence of a difference between the two groups in terms of the ideal-CDRS (i.e., the mean score was 3.61 in both groups). We also found a significant main effect of the type of CDRS, *F*(1, 62) = 6.90, *p* < .05, \eta _p^2 = .10. Overall, the scores on the actual-CDRS were higher than the scores on the ideal-CDRS, 4.98 vs. 6.31. The main effect of group was unreliable, *F* < 1.

#### BMI

The mean BMI was lower in participants who were low in body dissatisfaction as compared to participants who were high in body dissatisfaction, 20.17 vs. 23.14, respectively, *t*(62) = 4.22, *p* < .001, *d* = 1.05.

### Correlational Analyses

Table 1 provides an overview of the descriptive statistics of and pairwise correlations between the RRT scores, the CDRS scores of actual and ideal body image, explicit body dissatisfaction as measured by the EDI, the ratings of the RRT statements, and the BMI. Due to our sampling method (i.e., two groups with either a high or a low level of body dissatisfaction), most variables were not normally distributed. Accordingly, Spearman’s rank order correlations were computed. The actual-RRT correlated significantly with the explicit measures of actual body image (i.e., actual-CDRS scores, actual-RRT statement ratings, and BMI scores), but not with the explicit measures of ideal body image (i.e., ideal-CDRS scores and ideal-RRT statement ratings). The ideal-RRT, in contrast, correlated significantly with Ideal-CDRS scores, but was unrelated to explicit measures of actual body image (i.e., actual-CDRS scores, actual-RRT statement ratings, and BMI scores). Finally, it was observed that the actual-RRT and the ideal-RRT did not correlate with one another.

### Hierarchical Logistic Regression Analyses

To further validate the hypothesis that the two RRTs captured different implicit beliefs, a logistic regression was performed in which the two RRT scores as well as their interaction were used as predictors of whether a participant had been assigned to the group with a high or low degree of body dissatisfaction. On the basis of the full model, 67.2 % of all participants were classified correctly, *χ*^2^(3) = 12.98, *p* < .001, Nagelkerke *R*^2^ = .25. Whereas the actual-RRT contributed significantly to the prediction of group membership, *Wald* = 7.32, *p* < .05, *OR* = 0.42, the effects for the ideal-RRT and the interaction term were marginally significant only, *Wald* = 2.83, *p* = .09, *OR* = 1.69, and *Wald* = 2.97, *p* = .09, *OR* = 0.58, respectively.

Subsequently, we examined the predictive validity of the RRT data over and above the predictive validity of the (mean) explicit ratings of the statements used in the two RRTs. In a first step, the rating scores for the actual-RRT, the rating scores for the ideal-RRT, as well as the interaction between the two ratings scores were used as predictors of group membership. Results showed that the rating scores for the actual-RRT were highly predictive of group membership, *Wald* = 5.24, *p* = .05, *OR* = 0.011, whereas the other predictors were not (i.e., *Wald* < 1.80, *p*s > .18). Accordingly, only the rating scores for the actual-RRT were retained for a hierarchical analysis in which the two RRT scores and their interaction were entered in a second step. While the overall model fit was very high, *χ*^2^(4) = 67.75, *p* < .001, Nagelkerke *R*^2^ = .87 (93.8 % correct classifications), the added value of the RRT measures was negligible, *χ*^2^(3) = 2.75, *p* > .40.

A similar analysis was performed for the CDRS measures. Both the actual-CDRS scores and the ideal-CDRS scores were good predictors of group membership, *Wald* = 16.68, *p* < .001, *OR* = 39.69, and *Wald* = 8.82, *p* < .005, *OR* = 0.12. In contrast, the interaction between the two CDRS measures was unrelated to group membership, *Wald* < 1.76, *p* > .18, and was thus dropped from the model. When adding the two RRT measures and their interaction in a second step, model fit increased to a significant extent, *χ*^2^(3) = 8.48, *p* < .05. Final results showed, however, that only the actual-RRT score tended to be predictive of group membership over and above to the two CDRS measures, *Wald* = 3.80, *p* = .051, *OR* = 0.36. On the basis of the full model, 90.6 % of all participants were classified correctly, *χ*^2^(5) = 55.17, *p* < .001, Nagelkerke *R*^2^ = .77.

Finally, we examined the predictive validity of the RRT measures over and above the BMI. While the BMI alone was a reliable predictor of group membership, *Wald* = 10.72, *p* < .05, *OR* = 3.83, the inclusion of the two RRT measures and their interaction resulted in a significantly better model fit, *χ*^2^(3) = 10.35, *p* < .05. Whereas the ideal-RRT contributed significantly to the prediction of group membership, *Wald* = 4.44, *p* < .05, *OR* = 2.34, the effects for the actual-RRT and the interaction term just missed significance, *Wald* = 2.79, *p* = .10, *OR* = 0.55, and *Wald* = 3.71, *p* = .05, *OR* = 0.48, respectively. On the basis of the full model, 76.6 % of all participants were classified correctly, *χ*^2^(4) = 27.17, *p* < .001, Nagelkerke *R*^2^ = .46.

## Discussion

The degree to which people are dissatisfied with their own body is assumed to reflect a (perceived) discrepancy between the actual and the ideal body image (e.g., Cooper & Taylor, 1988; Strauman et al., 1991; Williamson et al., 1993). Consistent with this idea, Heider et al. (2015) observed that implicit beliefs about actual and ideal body image, as measured by the IRAP, were different in participants who reported either a low or a high degree of body dissatisfaction. In the present study, using the RRT (De Houwer et al., 2015) as a measure to capture implicit beliefs, we replicated the findings by Heider et al. (2015). Specifically, we observed that the implicit belief to be thin was more pronounced for participants low in body dissatisfaction as compared to participants high in body dissatisfaction. The implicit desire to be thin, in contrast, tended to be more pronounced in participants high in body dissatisfaction as compared to participants low in body dissatisfaction. These findings demonstrate that the RRT, like the IRAP, is capable of distinguishing between closely related beliefs that differ only in their relational component (i.e., ‘I am thin’ vs. ‘I want to be thin’). Additional findings corroborated this inference. The actual-RRT correlated with an explicit measure of actual body image but not with an explicit measure of ideal body image. Conversely, the ideal-RRT correlated with an explicit measure of ideal body image but not with an explicit measure of actual body image. Taken together with the observation that both RRTs were unrelated to each other, these findings strongly support the conclusion that both RRT measures, despite their structural similarity, captured different beliefs.

The ability of the RRT to pick up inter-individual differences in implicit beliefs is an important finding as complex relational knowledge is rather difficult to capture using more traditional implicit measures such as the IAT (Greenwald et al., 1998). In the context of body dissatisfaction, for example, one might construct an IAT in which words referring to the self or well-known other persons are either mapped on the same response key or a different response key as words referring to thinness and overweight (i.e., Bluemke & Friese, 2012). While such an approach would allow for an assessment of the degree to which the self is linked in memory to the concept of thinness/thickness, it would remain unknown whether this link reflects and actual state (i.e., ‘I am thin’) or a desired state (i.e., ‘I want to be thin’). If such a discrimination is the objective, relational implicit measures are needed. The present data thus show that the RRT can be a valuable addition to the traditional toolbox of implicit measures, especially in the field of body dissatisfaction.

**Figure 1:**
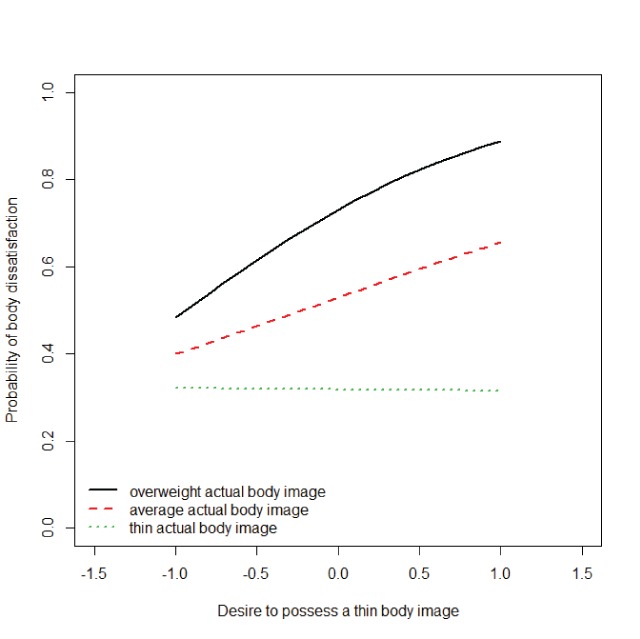
Probability of group membership (high vs. low degree of body dissatisfaction) as a function of the implicit desire to possess a thin body (i.e., D1 scores for the ideal-RRT), separated for low (i.e., 1 SD below average), average, and high (i.e., 1 SD above average) degrees of the implicit belief that one is thin (i.e., D1 scores for the actual-RRT).

The present results also go beyond the findings of Heider et al. (2015), in several ways. First, while Heider et al. (2015) observed no incremental predictive validity of the IRAP over and above explicit measures of body dissatisfaction, we observed that the predictive validity RRT measures were predictive of body dissatisfaction over and above the CDRS and the BMI. This observation is important as it suggests that the RRT measures developed here may eventually be used to predict important behavioral outcomes (e.g., relapse in eating disorders). Still, the observation that there was no evidence of incremental predictive validity over and above the explicit ratings is somewhat problematic from this perspective. It should be noted, however, that implicit measures are expected to be particularly useful in situations where explicit measures are biased as the result of social desirability or self-presentation concerns. It could thus be hypothesized that the added value of the RRT scores over and above the explicit ratings might surface in participants who are motivated to respond in a biased manner. Second, while Heider et al. (2015) simply observed a correlation between body dissatisfaction and the scores of the ideal-IRAP, the present data suggest that the degree to which the implicit desire to be thin is predictive of body dissatisfaction might be dependent on the extent to which one beliefs to be thin at the implicit level. As can be seen in Figure 1, for participants whose actual-RRT score was indicative of the belief to be overweight, body dissatisfaction increased as the implicit desire to be thin increased. In contrast, participants whose actual-RRT scores were indicative of the belief to be thin, the implicit desire to be thin was unrelated to group membership. This data pattern is in perfect accordance with the conceptualization of body dissatisfaction as the (self-perceived) discrepancy between actual and ideal body image. According to such a viewpoint, the desire to be thin should promote body dissatisfaction only if a person does not possess the belief of being thin. It must be noted, however, that the interaction between implicit actual and ideal body image just missed significance, so some caution is advised in drawing these conclusions.

The present study already has important practical implications. Albeit the internal consistency estimates for the actual-RRT and the ideal-RRT were relatively modest (*Rsb* = 0.49 and *Rsb* = 0.57, respectively), they were clearly much higher than those reported by Heider et al. (2015) for the actual-IRAP and the ideal-IRAP (*Rsb* = 0.32 and *Rsb* = 0.24, respectively). In addition, in both RRTs, the mean response latency was well below 2000 ms and the overall error rate was smaller than 15 %. Finally, less than 6 % of the participants were outliers in terms of their mean speed of responding or error rate and the overall results were unaffected by the inclusion or exclusion of these participants. Taken together, these observations add further weight to the idea that, in comparison to the IRAP, the RRT is an easy-to-complete instrument that might be useful as a diagnostic instrument also outside of the laboratory.

It may be noted, however, that more research would be needed to substantiate the causal nature of the relationship between, on the one hand, implicit beliefs about actual and ideal body image and, on the other hand, important behavioral outcomes such as the occurrence or maintenance of eating disorders. As an experimental approach rather than a correlational approach is needed to address this issue, it seems particularly interesting to start examining how and to what extent (complex) implicit beliefs can be changed. Not only would such an approach advance our general understanding of the relationship between implicit beliefs and behavioral outcomes, it is also likely to result in new therapeutic intervention strategies.

## Additional Files

The additional files for this article can be found as follows:

10.5334/pb.362.s1 Appendix [Description].
