# Supplementary material for: Body Dissatisfaction Revisited: On the Importance of Implicit Beliefs about Actual and Ideal Body Image
Source: Psychol Belg. 2018 Jan 4;57(4):158–73. doi: 10.5334/pb.362 (PMC6194529; doi:10.5334/pb.362)
Supplement: Appendix — [Description]. [file pb-57-4-362-s1.pdf]

Appendix

Table 2. Inducer words used in the RRTs

Inducer word	English translations	Inducer word	English translations
goed	good	mis	wrong
juist	right	onjuist	incorrect
correct	correct	incorrect	incorrect
exact	exact	verkeerd	wrong
in orde	alright	fout	error

Table 3. Target statements used the actual-RRT

Target statements	English translations
Ik heb een tengere lichaamsbouw.	I have a slender physique.
Ik weeg weinig.	I weigh little.
Ik zie mezelf als een slank persoon.	I consider myself a slim person.
Ik heb een mager figuur.	I have a lean figure.
Ik ben fijngebouwd.	I am delicately built.
Ik heb geen zware lichaamsbouw.	I do not have a fat physique.
Ik weeg niet te veel.	I do not weigh too much.
Ik zie mezelf niet als een dik persoon.	I do not consider myself a fat person.
Ik heb geen mollig figuur.	I do not have a chubby figure.
Ik ben niet struis.	I am not robustly built.
Ik heb geen tengere lichaamsbouw.	I do not have a slender physique.
Ik weeg niet te weinig.	I do not weigh too little.
Ik zie mezelf niet als een slank persoon.	I do not consider myself a slim person.
Ik heb geen mager figuur.	I do not have a lean figure.
Ik ben niet fijngebouwd.	I am not delicately built.
Ik heb een zware lichaamsbouw.	I have a fat physique.
Ik weeg te veel.	I weigh too much.
Ik zie mezelf als een dik persoon.	I consider myself a fat person.
Ik heb een mollig figuur.	I have a chubby figure.
Ik ben struis.	I am robustly built.

Table 4. Target statements of the ideal-RRT

Target statements	English translations
Ik wil een meer tengere lichaamsbouw hebben.	I want to have a more slender physique.
Ik streef ernaar minder te wegen.	I strive to weigh less.
Het is mijn wens om slanker te zijn.	It is my wish to be slimmer.
Ik wil een mager figuur hebben.	I want to have a leaner figure.
Ik wil fijner gebouwd zijn.	I want to be more delicately built.
Ik wil een minder zware lichaamsbouw hebben.	I want to have a less fat physique.
Ik streef er niet naar meer te wegen.	I don't strive to weigh more.
Het is niet mijn wens om dikker te zijn.	It is not my wish to be fatter.
Ik wil geen mollig figuur hebben.	I don't want to have a chubbier figure.
Ik wil minder struis zijn.	I want to be less robustly built.
Ik wil een minder tengere lichaamsbouw hebben.	I want to have a less slender physique.
Ik streef er niet naar minder te wegen.	I don't strive to weigh less.
Het is mijn wens om minder slank te zijn.	It is my wish to be less slim.
Ik wil geen mager figuur hebben.	I don't want to have a leaner figure.
Ik wil minder fijn gebouwd zijn.	I want to be less delicately built.
Ik wil een zwaardere lichaamsbouw hebben.	I want to have a fattier physique.
Ik streef ernaar meer te wegen.	I strive to weigh more.
Het is mijn wens om dikker te zijn.	It is my wish to be fatter.
Ik wil een mollig figuur hebben.	I want to have chubbier figure.
Ik wil struiser zijn.	I want to be more robustly built.
